# Influence of Grape Pomace Intake on Nutritional Value, Lipid Oxidation and Volatile Profile of Poultry Meat

**DOI:** 10.3390/foods9040508

**Published:** 2020-04-17

**Authors:** Francesca Bennato, Alessio Di Luca, Camillo Martino, Andrea Ianni, Elettra Marone, Lisa Grotta, Solange Ramazzotti, Angelo Cichelli, Giuseppe Martino

**Affiliations:** 1 Faculty of BioScience and Technology for Food, Agriculture and Environment, University of Teramo, Via Renato Balzarini 1, 64100 Teramo, Italy; fbennato@unite.it (F.B.); alessiodiluca@gmail.com (A.D.L.); aianni@unite.it (A.I.); emarone@unite.it (E.M.); lgrotta@unite.it (L.G.); sramazzotti@unite.it (S.R.); 2 Istituto Zooprofilattico Sperimentale dell’Abruzzo e del Molise “G. Caporale”, Via Campo Boario 37, 64100 Teramo, Italy; c.martino@izs.it; 3 Department of Medical and Oral Sciences and Biotechnologies, D’Annunzio University of Chieti-Pescara, 66100 Chieti, Italy; angelo.cichelli@unich.it

**Keywords:** grape pomace, broiler meat, linoleic acid, lipid oxidation, volatile compound, biogenic amines

## Abstract

Grape pomace (GP) represents the main solid by-product deriving from grape processing. The aim of this study was to evaluate the effect of dietary GP intake on nutritional quality, lipid oxidation and volatile profile of chicken meat. A total of 112 Ross 508 broilers were randomly divided into 4 groups and fed for 21 days with a standard diet. For the remaining 28 days of the trial, the control group (CG) continued to receive a standard diet, while the experimental groups (EGs) were fed with diets containing different GP concentrations: 2.5% (EG1), 5% (EG2) and 7% (EG3). Following the slaughtering, samples of breast meat were collected from each group. No significant differences were observed for pH, cooking loss and meat brightness, whereas the GP intake showed effectiveness in inducing variations in drip loss, meat yellowness and redness. The experimental feeding strategy also induced changes in the fatty acid profile, with an overall increase in polyunsaturated fatty acids (PUFA), mainly due to the increase in concentration of linoleic acid. The dietary supplementation also induced a decrease in lipid oxidation in meat, a finding also confirmed by the reduction in volatile aldehydes in 7 days stored raw meat. The feeding strategy based on the use of GP did not induce detrimental effects on the quality of broiler meat and showed the potential to lengthen the shelf-life as a direct consequence of the improvement in the oxidative stability. Overall, the present study showed a viable way for the recovery and the valorization of an agro-industrial by-product, with potential benefits also from an environmental point of view.

## 1. Introduction

The International Organization of Vine and Wine (OIV) reported for 2018 a global grape production equal to 78 million tons. Approximately, 50–75% of the grapes are used for juice production or winemaking, with the consequent accumulation of a significant amount of semisolid and hardly degradable residues accounting for about the 20% of the total processed grapes. Such residues are commonly used as fertilizer or simply discarded and, as for all agro-industrial by-products, their management represents an issue of great importance for its environmental and economic impact. Grape pomace (GP) represents the main solid by-product of the wine industry and, over time, numerous studies have been conducted with the aim of characterizing this matrix, which is particularly rich in compounds with considerable biological value [1]. The nutraceutical potential of many of these compounds, in particular polyphenols, has been associated with remarkable health benefits for humans, especially in the prevention and treatment of several chronic diseases [2]. In view of the above, GP received increasing attention in the zootechnical sphere as an alternative feed ingredient for livestock. In this regard, numerous studies have been conducted in recent decades, in particular on ruminants, highlighting many advantages of dietary GP intake on chemical and nutraceutical properties of milk and derived cheeses [3].

The use of agro-industrial by-products as dietary supplements for farm animals has shown on the whole significant advantages associated with qualitative characteristics of animal productions, as well as positive repercussions on animal welfare. For instance, the exploitation as zootechnical feed of olive pomace, resulting from the processing necessary for olive oil production, has been reported to improve the qualitative parameters associated with cow’s milk and its derived cheeses [4], while in the case of laying hens, positive effects have been evidenced with regard to inflammatory processes and the regulation of cholesterol biosynthesis [4,5].

With specific reference to the use of GP, several studies have been conducted on dairy ruminants, through which has been highlighted the potential of this by-product in inducing an improvement in the chemical-nutritional qualities of animal productions, as well as the sensory parameters as a consequence of the development, especially in ripened dairy products, of pleasant and highly appreciated aromatic notes [6,7]. The analysis of the whole blood transcriptome in Friesian calves showed also for dietary GP supplementation, the ability to positively modulate the pathway of cholesterol biosynthesis and antioxidant response, a finding that was associated with the significant decrease in concentration of serum cholesterol and an improvement in the oxidative stability in the carcasses [8]. In a study conducted by Gómez-Cortés et al. [9], GP has been used as dietary supplement for dairy ewes, and an in-depth study has been performed on the quality of meat samples obtained from their suckling lambs, without highlighting negative effects on carcasses characteristics, but evidencing an improvement in the water holding capacity. The GP addition to calves diet resulted instead in the increase in concentration of linoleic acid in meat samples from *longissimus dorsi*, contributing to the raising of the ratio between polyunsaturated fatty acids (PUFA) and saturated fatty acids (SFA). Furthermore, also in this condition, a reduction in lipid oxidation in stored raw meat was observed, as a presumable consequence of the antioxidant activity performed by bioactive compounds taken by diet [10]. Furthermore, the metagenomic analysis of rumen liquor in dairy calves, showed dietary GP to induce a taxonomic enrichment, mainly due to the increase in *Ruminiclostridium* and *Eubacterium* sp., whose metabolic activity was associated with the degradation of GP constituents, such as flavonoids and xyloglucan [11]. With specific regard to broilers, Aditya et al. [12] reported dietary GP to induce an improvement in meat quality, without experiencing detrimental effects on growth performance and carcass traits.

A growing interest has therefore developed around the GP exploitation as an alternative ingredient for zootechnical use, mostly justified by the encouraging results obtained in the dairy sector. Less attention has however been given to meat products, and in particular for the poultry sector, there is the need for further information that may characterize the potential role of this by-product in influencing qualitative variations. Therefore, this experimentation has been reasoned for the purpose to focus more attention on poultry products in order to deeply investigate the dietary GP effects on meat quality, oxidative stability, volatile compounds release and trace compounds accumulation.

## 2. Materials and Methods

This study was performed in a commercial farm and animals were handled following the national legislation on animal welfare (DL n. 126, 07/07/2011, EC Directive 2008/119/EC), and then slaughtered in compliance with Regulation 1099/2009 of the European Union on the protection of animals at the time of killing. For the scope of the study, animals did not undergo breeding practices other than those commonly adopted, and for this reason it is not considered necessary to provide further ethical declarations.

### 2.1. Animals Management and Sampling

The study was conducted in a poultry farm located in central Italy (Casoli, CH, Abruzzo). A total of 112 Ross 508 male chickens were randomly assigned to four groups (28 animals for each group) and for the first 24 days of the production cycle, all animals received a standard diet. For the remaining 25 days of the trial, the control group (CG) continued to receive a standard diet, while the experimental groups (EGs) were fed diets containing different GP concentrations: 2.5% (EG1), 5% (EG2) and 7% (EG3) on a dry matter basis. Specifically, chickens were fed across three different isoproteic and isoenergetic diets, with a changing basal composition: starter (0–10 days), grower (11–24 days) and finisher (25–49 days). The diets composition was defined taking into account the nutritional requirements indicated by the National Research Council (NRC) [13]. Ingredients and chemical composition of finisher diets administered to CG, EG1, EG2 and EG3 chickens have been reported in Supplementary Table S1. Access to feed and water was provided ad libitum for the entire duration of the production cycle. From the beginning of the trial and during the 49 days of the experimentation, the feed intake (FI) and the body weight (BW) were weekly calculated for each group of animals, for a total of 8 evaluations, which were useful for the determination of the feed conversion ratio (FCR; cumulative feed intake/body weight). Data concerning BW and FCR are reported in Supplementary Table S2. The GP used in the study derived from red grape (*Vitis vinifera* L.) and was obtained as previously described [10]. Briefly, the GP, consisting of peels, seeds and a small amount of stems, was collected from a local vineyard; following distillation for alcohol extraction and treatment with water at 90 °C in order to recover the tartaric acid, the leftover was dried and properly preserved until the start of the trial.

At the end of the experimentation, all chickens were slaughtered in a commercial abattoir. The carcass of each animal was weighted and the carcass yield was calculated by taking into account the previously performed evaluations of the live weight. Carcasses were then left at a controlled temperature of 4 °C, covered by a synthetic film in order to avoid the exposure to the surrounding environment. After 24 hours, the hemibreast of each chicken was collected, and in order to highlight variations on raw meat quality due to the storage at 4 °C, the sampling was performed after 3 (T_3_) and 7 (T_7_) days post mortem, paying attention every time to eliminate approximately 0.3–0.5 cm of the most exposed layer of meat before performing the sampling.

The evaluations described below concerning color, chemical composition, ability of raw and cooked samples to retain water (drip loss and cooking loss respectively) and fatty acid profile, were carried out only on samples collected 3 days after the date of slaughter, while the lipid oxidation, the release of volatile compounds, and the accumulation of biogenic amines were evaluated both after 3 and 7 days post mortem. The analysis concerning color, drip loss and cooking loss were performed on fresh samples. The remaining evaluations (fatty acid profile, lipid oxidation, release of volatile compounds and accumulation of biogenic amines) were instead performed on meat that after sampling was stored under vacuum at −20 °C.

### 2.2. Evaluation of pH and Color

The pH evaluation on chicken breast samples 48 h (pH_48_) after slaughtering was performed by using a portable pH meter equipped with an electrode (Crison, Barcelona, Spain) that was inserted about 1.0–1.5 cm into the tissue, adjusting each evaluation in relation to the muscle temperature. Before the analysis, a calibration of the instrument was performed by using standard phosphate buffers (pH 4.00 and 7.00) and at the end of each measurement the electrode was carefully rinsed in distilled water before the next evaluation.

Color measurements were performed on the transverse section of the chicken breast muscle by using the Minolta-CR 300 (Minolta Co, Osaka, Japan), with a D65 illuminant, 10 standard observer angle and 30 mm of aperture size. All evaluations were carried out taking into account the CIELAB system, which exploits the chromaticity coordinates L* (lightness), a* (redness) and b* (yellowness). Before each series of measurements, the colorimeter was calibrated by using a white tile (L* = 100) and black glass (L* = 0).

### 2.3. Drip Loss, Cooking Loss, and Chemical Composition of Meat Samples

The drip loss was performed in order to determine the raw meat’s tendency to retain water. Samples with a thickness of 2.0–2.5 cm and approximate weight of 75 g were inserted in an expanded bag and left hanging at 4 °C, avoiding the contact of the meat with the wall of the container. After 24 h, all samples were re-weighed after drying the surface of each one with absorbent paper. Drip loss was then reported as a percentage of the initial sample weight.

The cooking loss, useful to characterize the ability of meat to retain water during cooking, was evaluated on T_3_ samples. Meat portions of 75–80 g and an approximate thickness of 2.5 cm were cooked in a water bath (Grant Instruments Ltd., Barrington, UK) until the core temperature of 70 °C (Minitherm HI8751 temperature meter and probe, Hanna Instruments Ltd., Bedfordshire, UK) was reached. Samples were then cooled to room temperature before being stored overnight at 4 °C. After weighing the cooked samples, the cooking loss was expressed as a percentage of the initial raw sample weight.

The evaluation of meat chemical composition has been made in reference to the protocols of the Association of Official Analytical Chemistry (AOAC) [14]. Specifically, meat samples were preventively ground, then analyzed in order to obtain information on moisture, proteins, fat and ash content.

### 2.4. Fatty Acid Composition and Lipid Oxidation

The evaluation of the fatty acid composition in GP administered to the CG and EGs was performed following the procedure reported by Castellani et al. [4].

With regard to the meat samples, the extraction of total lipids was performed by using the Folch method [15]. Approximately 4 g of preventively minced meat were homogenized in 72 mL of Folch solution by using an Ultra-Turrax T-25 (Janke & Kunkel & Co. IKA Labortechnik, Staufen, Germany). After stirring for 6 h at room temperature, all samples were filtered overnight in a sodium chloride solution. Total fat for each sample was obtained through the chloroform phase evaporation to dryness by using a Strike-Rotating Evaporator (Steroglass S.r.l., Perugia, Italy) set at 38 °C. For each sample, the formation of fatty acid methyl esters (FAME) was induced by mixing 60 mg of fat with 5 mL of hexane containing 100 µL of sodium methoxide and 100 µL of methanol. FAMEs detection was performed by a gas chromatograph (Focus GC; Thermo Scientific, Waltham, MA, USA) equipped with a flame ionization detector (FID) and a capillary column (Restek Rt-2560 Column fused silica 100 m 0.25 mm highly polar phase; Restek Corporation, Bellefonte, PA, USA). The ChromeCard software was used for quantification of the peak areas, and the relative values associated with individual FAs were expressed as a percentage of total FAME. For the identification of individual FAMEs were exploited the retention times recorded with the standard mixture FIM-FAME7-Mix and individual trans 11 C18:1 (Matreya LLC, State College, PA, USA).

Lipid oxidation was evaluated through the method of thiobarbituric acid reactive substances (TBARS), following the procedure previously described by Ianni et al. [16].

### 2.5. Volatile Compounds Evaluation

Five grams of preventively minced meat were weighed and mixed with 10 mL of an aqueous solution of saturated NaCl (360 g/L). Following a slight homogenization with Ultra-Turrax T-25 (6500 rpm for 15 sec; Janke & Kunkel & Co. IKA Labortechnik, Staufen, Germany) 10 µL of the internal standard solution (3-methyl-2-heptanone; 10 µg/L in ethanol) were added. A solid-phase microextraction fiber (divinylbenzene-carboxen-polydimethylsiloxane; length: 1 cm; film thickness: 50/30 m; Sigma-Aldrich, Milan, Italy) was used to perform the headspace extraction of volatile compounds (VOCs) with an exposition time of 45 min at 55 °C. The extracted VOCs were then thermally desorbed into a Clarus 580 gas chromatograph (Perkin Elmer, Waltham, MA, USA) equipped with an Elite-5MS column (length internal diameter: 30 × 0.25 mm; film thickness: 0.25 µm; Perkin Elmer, Waltham, MA, USA) and coupled with a mass spectrometer (SQ8S; Perkin Elmer, Waltham, MA, USA). The setting concerning the thermal program and the VOCs identification were performed as previously described [17].

### 2.6. High Performance Liquid Chromatography for the Analysis of Biogenic Amines

High performance liquid chromatography (HPLC) was used to evaluate the accumulation of biogenic amines on the meat samples. Raw meat samples were previously subjected to extraction and derivatization by using dansyl chloride, then the analysis was performed in accordance with the method described by Schirone et al. [18] with slight modifications related to the applied elution gradient; the whole procedure is briefly described below. The chromatographic apparatus was constituted by a HPLC (Varian, Palo Alto, CA, USA) equipped with a Supelcosil LC-18 column (25 cm × 4.6 mm, 5 m; Supelco) and connected to an UV-VIS wavelength detector. The mobile phase prepared for amines separation was constituted by ultrapure water (A) and acetonitrile (B). At the beginning of the analysis, A represented 50% of the mobile phase, and this percentage underwent a linear reduction of up to 10% in 20 min; then, the A content increased up to 50% in 5 min and this condition was held for 10 min until the end. A flow rate of 1 mL/min was applied and the column temperature was set at 38 ± 0.1 °C. The chromatographic peaks were detected at a fixed wavelength of 254 nm and the identification of the biogenic amines was performed, taking into account the retention times. The calibration curves (peak area versus concentration) were obtained by using tyramine, putrescine, cadaverine, tryptamine, 2-phenethylamine and serotonin. Such curves were linear in the range of concentration between 0.5 and 50 mg/L, and the amount of each analyte was determined by using the method of the internal standard (1,7-diaminoheptane), through interpolation of regression lines.

### 2.7. Statistical Analysis

All the described evaluations were performed on 15 animals (randomly selected) per group, and the analysis on the single sample was performed in triplicate. Results were reported as mean values with the correspondent standard deviations (SD). The data were checked for normality distribution and analyzed by one-way ANOVA, using the GLM Procedure [19] of the statistical package SPSS 13.0 (SPSS Inc., Chicago., IL) for statistical analyses considering the effect of food treatment as a factor of variation. When interactions were observed (*p* ≤ 0.05), Tukey’s honest significance test was used to compare treatment means.

## 3. Results

### 3.1. Physical and Chemical Characterization of Chicken Breast Meat

The dietary GP supplementation did not induce in chicken breast meat samples variations in pH measured after 48 h post mortem and in the ability to retain water during cooking (*p* > 0.05). As reported in Table 1, the drip loss showed instead a significant increase in water loss (*p* < 0.05) in EG2 and EG3 samples, deriving from animals fed with the higher GP concentration, 5% and 7% respectively.

With regard to the meat color, no variations were highlighted for lightness (L*), while significant changes characterized redness (a*) and yellowness (b*). In particular, the dietary GP intake induced an increase in both chromaticity coordinates a* and b* (*p* < 0.01). However, it is noteworthy that no significant chromatic variations were observed within the groups that received the experimental diets (EG1, EG2 and EG3).

In Table 1, the data obtained from chemical characterization of chicken breast meat are also shown. In this context, the only difference was associated with the amount of total lipids which increased in concentration, although not significantly (*p* = 0.056), in EG2 and EG3 samples obtained from broilers that respectively received the 5% and 7% of dietary GP supplementation.

### 3.2. Fatty Acid Composition and Lipid Oxidative Stability

The characterization of the fatty acid composition in GP showed linoleic acid (C18:2 *cis*-9 *cis*-12) to be the most represented, accounting for 66.45% ± 4.78% of the total fatty acids identified in the agro-industrial by-product, followed by 14.61% ± 1.31% of oleic acid (C18:1 *cis*-9), 10.83% ± 0.86% of palmitic acid (C16:0), 3.16% ± 0.28% of stearic acid (C18:0) and 2.22% ± 0.23% of linolenic acid (C18:3 *cis*-9 *cis*-12 *cis*-15).

The evaluation of the fatty acid profile on chicken meat (Table 2) showed only a significant increase (*p* < 0.05) in concentration of linoleic acid in EG2 and EG3 samples, respectively deriving from animals fed the 5% and 7% dietary supplementation with GP. The dietary intake also induced a reduction in SFA, which was significant (*p* < 0.05) for EG2 and EG3 samples in comparison with CG samples.

Besides C18:2, the only PUFA identified in the meat was C18:3, which did not undergo changes as a consequence of the dietary supplementation. However, the increased amount of C18:2 in EG2 and EG3 samples was effective in inducing an increase in total PUFA (*p* < 0.05), a finding also responsible for the increased PUFA/SFA ratio in the same samples. No significant changes were instead reported for MUFA (*p* > 0.05).

**Figure 1:**
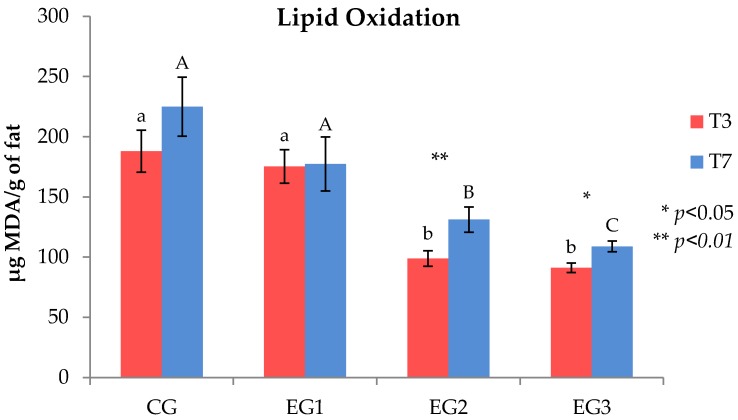
Oxidation profile of T_3_ and T_7_ raw meat samples obtained from chicken fed the standard diet (CG) and chicken fed the dietary grape pomace supplementation of 2.5% (EG1), 5% (EG2) and 7% (EG3). Data are reported as µg of malondialdehyde (MDA) per g of fat. Red bars represent the values related to T_3_ samples, while the blue bars represent the values of samples stored up to 7 days (T_7_) at 4 °C. Different lower case letters (a, b) indicate significant differences between T_3_ samples (*p* < 0.05), while upper case letters (A, B, C) indicate significant differences between T_7_ samples (*p* < 0.05). Significant differences within the same group are indicated with asterisks (* *p* < 0.05, ** *p* < 0.01).

The TBARS test (Figure 1) showed the dietary GP supplementation to reduce the lipid oxidation in a dose dependent manner. EG1 meat samples, collected both at T_3_ and T_7_, did not show significant differences in comparison to CG samples, however a reduction in MDA release was noted. On the other hand, with regard to the EG2 and EG3 samples, a significantly lower degree of oxidation was observed with respect both to meat obtained from animals fed the standard diet (CG) and chicken fed the lower dietary GP supplementation (2.5% on dry matter basis). Furthermore, for EG2 and EG3, it should be also evidenced the higher MDA concentration in T_7_ samples compared to T_3_ samples (*p* < 0.01 and *p* < 0.05 for EG2 and EG3 respectively).

### 3.3. Identification of Volatile Compounds in Chicken Meat

The evaluation of volatile profile in the raw chicken meat led to the identification of 14 compounds mainly belonging to the family of aldehydes and alcohols. However, only results obtained for T_7_ samples are shown (Table 3), since no variations were evidenced for T_3_ samples between the four analyzed groups.

The most represented compound is hexanal, which accounts for the 65.10% ± 5.01% of total VOCs in the CG and its concentration undergoes a significant reduction in the EG1, EG2 and EG3 samples (*p* < 0.05). Among aldehydes, an increase in octanal concentration was also recorded in samples obtained from chicken fed the 5% and 7% dietary GP supplementation (EG2 and EG3; *p* < 0.05). With specific regard to alcohols, the GP intake resulted effective in inducing an increase in concentration of 1-pentanol, 1-heptanol and 1-octanol. 1-pentanol and 1-heptanol were most represented in all experimental samples (*p* < 0.01) without significant differences between EG1, EG2 and EG3. The result is slightly more complex for 1-octanol, which showed a significant increase in EG1 meat with respect to the CG samples (*p* < 0.01). However, the concentration of this compound further increased in EG2 and EG3 raw meat compared to both the CG (*p* < 0.01) and EG1 (*p* < 0.05) samples. No significant changes were observed between groups for the only detected ketone, the 2,5-octanedione, and for benzaldehyde, an aromatic aldehyde (*p >* 0.05).

### 3.4. Evaluation of Biogenic Amines

The evaluation of biogenic amines through a HPLC approach was performed with the aim to evaluate in raw meat the presence of putrescine, cadaverine, tryptamine, 2-phenethylamine, tyramine and serotonin. In T_3_ samples, none of the listed compounds were found, whereas in samples stored for 7 days at 4 °C, only putresceins, cadaverins and tyramine were identified (Table 4), although their concentration was lower than the limit of quantification (LOQ).

## 4. Discussion

The present study was conducted with the aim to evaluate chemical-nutritional parameters of poultry meat obtained by feeding animals with three different dietary GP supplementations (2.5%, 5% and 7%, respectively). Particular attention has been given to the characterization of fatty acid profile, in addition to the evaluation of lipid oxidation, volatile profile and biogenic amines during meat storage at 4 °C. Prior to the listed analyses, basic zootechnical assessments associated with the periodic monitoring of chicken body weight, cumulative feed intake and, consequently, of the feed conversion ratio were performed. As reported in Table S2, these evaluations did not evidence significant variations between the groups of animals involved in the study. Therefore, this data testifies to the fact that the GP introduction in the animal diets had no effects on the amount of feed consumption, and that is in full agreement with what was previously reported [12].

The dietary GP intake did not affect the pH values measured in chicken breast muscle after 48 h post mortem (pH_48_) as well as cooking loss, for which no significant variation between the CG and experimental groups (EG1, EG2 and EG3) was observed. Changes were instead highlighted in the ability of raw meat to retain water (drip loss); in particular, EG2 and EG3 meat samples showed a greater weight loss compared to both the CG and EG1. The drip loss reflects the release of intramuscular components, mainly organic osmolytes, from muscle tissue. In a study conducted by Lambert et al. [20] has been characterized the release of organic osmolytes in a cellular model, C2C12 porcine muscle cells, exposed to anoxia and pH reduction from 7.4 to 6.0 in order to mimic the condition following the animal slaughter. Specifically, the authors reported a key role of 5-lipoxygenase in inducing drip loss. Several isoforms of this enzyme are particularly expressed in plant matrices [21,22], therefore they could have been taken by chickens with the diet, justifying the drip loss increase. However, it should be also mentioned that in grape derivatives, there have been isolated and characterized numerous compounds, above all polyphenols, credited with inhibitory function against 5-lipoxigenase [23]. For this reason, the observed finding deserves further and more specific assessments.

With regard to meat color, it should be considered that this parameter deserves attention as well as chemical assessments as it greatly influences the choice made by the consumer. In this study, significant differences have been evidenced for chromaticity coordinates a* and b* while no variations have been highlighted for L*. Specifically, the dietary GP supplementation showed to be effective in inducing a more intense red coloration and a concomitant shift of the color index towards yellow. Contrary to what was observed in this study, Kasapidou et al. [24] reported lower a* values with paler meat after feeding chicken with a dietary GP inclusion; furthermore, authors did not observe any significant differences in muscle lightness and yellowness. Such discrepancy can be justified, at least in part, by the high variability that is very often associated with this type of evaluation. In a study conducted by Lindahl et al. [25] on pork meat, it was evidenced that about the 86–90% of variations in meat lightness, redness, yellowness and chroma (saturation) can be explained by variations of pigment content, myoglobin forms and reflectance of internal surface. Specifically, lightness, redness and chroma appear to be influenced with similar extent by both the pigment content and the myoglobin forms, while yellowness seems to vary mostly in relation to the myoglobin forms, less to internal reflectance, without significant effects induced by the pigment content. In addition to what has been described, it should also be emphasized that the pigments content, especially anthocyanins, in grapes and its derivatives, can be significantly influenced by numerous factors, including grape variety and its dosage [26].

Concerning the chemical composition of meat samples, the dietary GP intake did not induce significant changes, confirming what has been previously described by Sáyago-Ayerdi et al. [27], who fed chickens with 0, 30 and 60 mg of a GP concentrate per kg of diet in the period between 3 and 6 weeks of age. This finding is also consistent with that reported for other animal species, as in the case of steers in the study conducted by Moote et al. [28].

In the last two decades, increasing interest has been focused on the development of feeding strategies useful to induce variations in the acidic profile of meat productions deriving from both ruminants and monogastric. The main reason behind this need lies in the fact that animal fats, with the exception of fish lipids, are generally characterized by marked concentrations of SFA, which are notoriously associated with several cardiovascular diseases [29]. This aspect acquires particular relevance in ruminants as a consequence of the ruminal biohydrogenation mechanism, which is responsible for dietary PUFA conversion into SFA or MUFA [30]. Additionally, in the case of broilers, this need is perceived, although the amount of fat present in this type of meat is generally limited (just over 1%), therefore any changes certainly have a lesser biological impact when compared to what happens in ruminants. In addition to this, it must be specified that the improvement in the health indexes of a food product is more specifically associated with the increase in omega-3 fatty acids. For this reason, several feeding strategies have been tested over time in order to obtain an enrichment of such compounds in animal products [31]. In this study, a significant increase in linoleic acid has been observed in meat samples obtained from animals that received the GP supplementation. This finding can be fully justified by the fact that linoleic acid has been reported to be the most represented fatty acid in grape pomace [3,32,33]. Therefore, a greater presence in the meat samples is presumably a function of its greater intake through the diet, a finding recently reported also in beef [10].

The marked increase in linoleic acid in the experimental samples also led to the significant overall increase in PUFAs. The presence of these compounds in food products is generally associated with a greater susceptibility to oxidative mechanisms, with potentially deleterious effects on conservation, safety, flavor and taste [29]. However, in this study, the TBARS test highlighted a greater oxidative stability of EG1, EG2 and EG3 meat samples in comparison with the CG samples. The dietary GP intake by chicken was therefore effective in preserving the lipid component from peroxidation. The enrichment of the animal diet with plant matrices is generally associated with an improvement in the oxidative stability of meat production, a factor well characterized for instance in the organic farming systems in which animals are left free to consume the plant resources present in the breeding area [34]. Such a finding has been already evidenced in chicken meat. Goñi et al. [35] reported that the use of feedstuffs rich in compounds credited with great value from a biological point of view, such as polyphenols, may reduce the intestinal vitamin E degradation, thus, allowing a greater quantity of this compound to be absorbed, consequently improving the oxidative stability in several tissues. A detailed study was conducted by Chedea et al. [36] in order to characterize the absorption and antioxidant activity of GP polyphenols in monogastrics, specifically weaned piglets. The in vivo approach showed the GP potential to improve the total antioxidant status in both duodenum and colon, with consequent decrease in lipid oxidation. These findings therefore support the hypothesis of a direct role of the GP bioactive compounds in improving the oxidative stability of meat products.

The role of volatile flavor compounds in meat has been widely investigated [37,38], and several studies paid attention to the possibility to induce variations in volatile profile of animal products by changing the feeding strategies [39], especially through the exploitation of vegetable matrices rich in biologically active compounds [10,40,41].

In order to obtain information more related to the diet administered to animals, in this study, the evaluation of volatile profile was performed on raw meat, contrary to most of the analyses that are usually performed on cooked samples with the aim to identify the VOCs generated by reactions induced by thermal processes [38]. Almost all of the compounds identified in chicken breast muscle belong to aldehydes and alcohols. Among aldehydes, the most represented compound was hexanal, which significantly decreased in samples obtained from animals fed the dietary GP supplementation. A feeding strategy characterized by high concentration of PUFA, and in particular of linoleic acid, has been reported in beef to correlate with a significant increase in aldehydes as a consequence of the presence of structures more susceptible to oxidative processes [42]. For this reason, the drastic reduction in hexanal release in EGs meat samples appears to be a contradiction, although this finding can be explained, at least in part, by the well-characterized antioxidant action performed by the bioactive compounds in which GP is particularly rich. In addition to this, the increase in hexanal concentration has been also reported to be one of the main causes for flavor deterioration during meat storage, especially in the early stages [43], giving further meaning to the observed data. The increase in concentration of 1-pentanol, 1-heptanol and 1-octanol in EG1, EG2 and EG3 chicken meat represents a finding difficult to comment on, since most of the investigations on meat volatile profile have been made on cooked products and not on raw meat. However, a useful cue can be obtained by taking into consideration the study of Ba et al. [44], who investigated the oxidative process leading to the VOCs release in meatlike models containing different unsaturated fatty acids, specifically oleic, linoleic and linolenic acids, in addition to amino acids and sugars (Maillard reactants). The authors evidenced the increase in concentration of 1-pentanol as a consequence of the oxidation resulting from the combination of amino acids, ribose and linoleic acid, while an increase in 1-octanol was registered following the oxidation of oleic acid and, in a less extent, by combining oleic acid with amino acids and ribose both at pH 5.5 and 6.2. Several factors are therefore able to influence such mechanisms and a precise characterization of the observed events would deserve dedicated evaluations.

The identification of biogenic amines in foods is a helpful approach for spoilage determination, since such compounds represent the result of several and well characterized metabolic processes of bacterial origin [45]. The most detected amines in meat are generally represented by histamine, cadaverine, tyramine and putrescine, each of which has different interaction capabilities with the biochemical mechanisms of the consumer [46]. In this study, only low concentrations of cadaverine, putrescine and tyramine were identified in samples stored for 7 days at 4 °C. Cadaverine was identified only in the CG samples, no variations were evidenced for tyramine, while a slight reduction was observed for putrescine in samples obtained from animals fed the GP supplementation. The presence of low concentrations of these compounds, especially on the exposed surfaces of food products, is commonly associated with proper handling [46] and the slight improvement in the finding observed in EGs samples could be related to the presumable action of some GP bioactive compounds credited with antimicrobial functions, therefore limiting microbial growth and biogenic amines accumulation [47]. However, it must also be said that all the values found are below the limit of quantification of the applied methodology, therefore it would be speculative to draw conclusions from the observed differences. In addition to this, no specific investigations have been performed on the potential activity of GP polyphenols on microbial metabolism, thus stimulating the need for further and more specific analyses.

## 5. Conclusions

Overall, it may be concluded that GP inclusion in poultry diet up to 7% is effective in inducing interesting variations in chicken meat quality. Our findings specifically suggest a positive role of GP in increasing the PUFA:SFA ratio as a direct consequence of the marked increase in concentration of linoleic acid. Furthermore, the improvement in the oxidative stability of poultry meat must be highlighted, a finding also confirmed by the significant decrease in volatile hexanal, a marker of lipid oxidation in food products. This aspect could therefore justify an extension of the shelf-life, with significant potential benefits for consumers’ health. In addition to this, the present study showed a practicable strategy for the recovery and valorization of the main by-product deriving from grape processing.

## Figures and Tables

**Table 1:** Physical and chemical characterization of the meat samples obtained from chicken fed the standard diet (CG) and chicken fed the dietary grape pomace supplementation of 2.5% (EG1), 5% (EG2) and 7% (EG3).

Trait		CG	EG1	EG2	EG3
pH_48_		5.93 ± 0.10	5.88 ± 0.15	6.04 ± 0.17	5.95 ± 0.12
Drip loss, %		2.30 ± 0.24 ^a^	2.35 ± 0.26 ^a^	2.60 ± 0.27 ^b^	2.89 ± 0.24 ^b^
Cooking loss, %		12.4 ± 1.41	10.4 ± 1.73	11.4 ± 1.92	11.7 ± 1.52
Chromaticity coordinates	L*	55.9 ± 3.22	54.6 ± 2.75	56.2 ± 2.29	54.2 ± 1.12
	a*	−2.37 ± 0.65 ^a^	−0.88 ± 0.16 ^b^	−1.07 ± 0.19 ^b^	−0.74 ± 0.11 ^b^
	b*	7.88 ± 0.89 ^a^	11.3 ± 1.46 ^b^	11.9 ± 1.27 ^b^	11.9 ± 1.81 ^b^
**Chemical composition, %**					
Moisture		74.4 ± 1.56	73.5 ± 1.10	73.5 ± 1.83	73.1 ± 1.17
Dry matter ^†^ (DM)		25.6 ± 1.56	26.5 ± 1.10	26.5 ± 1.83	26.9 ± 1.17
Total lipids ^†^		1.14 ± 0.14	1.16 ± 0.07	1.22 ± 0.17	1.25 ± 0.10
Proteins ^†^		23.4 ± 0.74	24.2 ± 1.04	23.8 ± 0.77	24.3 ± 1.13
Ash ^†^		1.08 ± 0.06	1.08 ± 0.05	1.02 ± 0.05	1.09 ± 0.02

All data are reported as mean ± standard deviation (SD); ^ab^ Different letters in the same row indicate significant difference (*p* < 0.05). pH_48_: pH 48 h post mortem. L*: lightness; a*: redness; b*: yellowness. ^†^ Data are reported on a dry matter basis.

**Table 2:** Fatty acid composition of the meat samples obtained from chicken fed the standard diet (CG) and chicken fed the dietary grape pomace supplementation of 2.5% (EG1), 5% (EG2) and 7% (EG3).

Fatty Acid ^†^	CG	EG1	EG2	EG3
C14:0	0.80 ± 0.10	0.66 ± 0.07	0.69 ± 0.09	0.72 ± 0.09
C14:1	0.07 ± 0.01	0.06 ± 0.01	0.06 ± 0.01	0.07 ± 0.01
C15:0	0.14 ± 0.02	0.11 ± 0.01	0.11 ± 0.02	0.16 ± 0.03
C16:0	26.6 ± 2.27	25.5 ± 1.16	24.1 ± 1.03	24.9 ± 1.89
C16:1	1.99 ± 0.24	2.02 ± 0.21	1.72 ± 0.18	2.15 ± 0.21
C17:0	0.36 ± 0.05	0.38 ± 0.03	0.36 ± 0.05	0.37 ± 0.06
C18:0	12.5 ± 0.74	12.9 ± 0.36	10.6 ± 0.52	10.9 ± 1.65
C18:1 *cis 9*	28.8 ± 2.36	28.8 ± 1.49	30.6 ± 2.22	29.5 ± 1.16
C18:2	22.6 ± 1.37 ^a^	23.7 ± 1.14 ^a^	25.7 ± 1.31 ^b^	25.8 ± 1.53 ^b^
C18:3	1.56 ± 0.17	1.58 ± 0.15	1.53 ± 0.24	1.66 ± 0.33
SFA	40.4 ± 1.82 ^a^	38.6 ± 1.20 ^ab^	35.8 ± 1.07 ^b^	37.1 ± 1.57 ^b^
MUFA	30.9 ± 2.65	30.9 ± 1.75	32.4 ± 2.06	31.8 ± 1.61
PUFA	24.2 ± 2.09 ^a^	26.3 ± 1.22 ^ab^	27.2 ± 1.48 ^b^	27.4 ± 1.28 ^b^
PUFA/SFA	0.60 ± 0.07 ^a^	0.68 ± 0.08 ^a^	0.76 ± 0.09 ^b^	0.74 ± 0.08 ^a,b^

^†^ Data are reported as mean percentage on total FAME ± standard deviation (SD). ^ab^ Different letters in the same row indicate significant difference (*p* < 0.05). SFA: saturated fatty acids (C14:0, C15:0, C16:0, C17:0, C18:0); MUFA: monounsaturated fatty acids (C14:1, C16:1, C18:1 *cis*9); PUFA: polyunsaturated fatty acids (C18:2, C18:3).

**Table 3:** Volatile profile of raw meat samples stored for 7 days at 4 °C and obtained from chicken fed the standard diet (CG) and chicken fed the dietary grape pomace supplementation of 2.5% (EG1), 5% (EG2) and 7% (EG3).

VOC ^†^	CG	EG1	EG2	EG3
Pentanal	1.67 ± 0.29	1.93 ± 0.31	2.28 ± 0.36	2.16 ± 0.31
Hexanal	65.1 ± 5.01 ^a^	56.8 ± 4.15 ^b^	54.0 ± 4.18 ^b^	56.9 ± 4.49 ^b^
Heptanal	3.64 ± 0.47	3.22 ± 0.41	3.86 ± 0.58	2.93 ± 0.42
Octanal	3.20 ± 0.43 ^a^	4.70 ± 0.52 ^a^	6.47 ± 0.72 ^b^	5.83 ± 0.62 ^b^
Nonanal	5.95 ± 0.63	6.64 ± 0.66	6.61 ± 0.60	6.74 ± 0.63
2-heptenal	0.77 ± 0.09	0.90 ± 0.15	0.62 ± 0.18	0.77 ± 0.11
2-octenal	0.61 ± 0.8	0.69 ± 0.07	0.63 ± 0.07	0.88 ± 0.11
1-pentanol	0.54 ± 0.08 ^a^	0.86 ± 0.10 ^b^	0.90 ± 0.12 ^b^	0.96 ± 0.12 ^b^
1-heptanol	0.26 ± 0.05 ^a^	1.56 ± 0.19 ^b^	1.75 ± 0.21 ^b^	1.62 ± 0.14 ^b^
1-octanol	0.49 ± 0.06 ^a^	1.13 ± 0.16 ^b^	1.59 ± 0.21 ^c^	1.53 ± 0.18 ^c^
1-octen-3-ol	9.24 ± 1.61	12.2 ± 1.95	12.6 ± 1.62	11.7 ± 1.38
2-octen-1-ol. (Z)-	0.69 ± 0.11	0.71 ± 0.09	0.67 ± 0.08	0.53 ± 0.07
2,5-octanedione	7.45 ± 0.77	8.14 ± 0.92	7.43 ± 0.58	7.03 ± 0.95
Benzaldehyde	0.39 ± 0.05	0.48 ± 0.07	0.56 ± 0.07	0.49 ± 0.08

^†^ Data are reported as mean percentage of each volatile compound (VOC) ± standard deviation (SD). ^abc^ Different letters in the same row indicate significant difference (*p* < 0.05).

**Table 4:** Biogenic amines detected in raw meat samples stored for 7 days at 4 °C and obtained from chicken fed the standard diet (CG) and chicken fed the dietary grape pomace supplementation of 2.5% (EG1), 5% (EG2) and 7% (EG3).

Biogenic Amine ^†^	CG	EG1	EG2	EG3
Putrescine	0.27 ± 0.04 ^a^	0.20 ± 0.03 ^b^	0.17 ± 0.02 ^b^	0.15 ± 0.01 ^b^
Cadaverine	0.24 ± 0.04	nd	nd	nd
Tyramine	0.18 ± 0.03	0.13 ± 0.05	0.20 ± 0.06	0.13 ± 0.04

^†^ Data are reported as mean (mg/L) ± standard deviation (SD). ^ab^ Different letters in the same row indicate significant difference (*p* < 0.05); nd: not detectable.

## Supplementary Materials

The following are available online at https://www.mdpi.com/2304-8158/9/4/508/s1, Table S1: Ingredients and chemical composition of finisher diets administered to chicken belonging to the control group (CG) and chicken fed the dietary grape pomace supplementation of 2.5% (EG1), 5% (EG2) and 7% (EG3). Table S2: Body weight (BW) and feed conversion ratio (FCR) evaluated after 1, 7, 14, 21, 28, 35, 42 and 49 days of age for chicken belonging to the control group (CG) and chicken fed the dietary grape pomace supplementation of 2.5% (EG1), 5% (EG2) and 7% (EG3).
