# Supplementary material for: Influence of Grape Pomace Intake on Nutritional Value, Lipid Oxidation and Volatile Profile of Poultry Meat
Source: Foods. 2020 Apr 17;9(4):508. doi: 10.3390/foods9040508 (PMC7230919; doi:10.3390/foods9040508)
Supplement: Supplementary file 1 [file foods-09-00508-s001.zip › Table S1.pdf]

Supplementary Table 1. Ingredients and chemical composition of finisher diets administered to chicken belonging to the control group (CG) and chicken fed the dietary grape pomace supplementation of 2.5 % (EG1), 5 % (EG2) and 7 % (EG3).

	Diet			
	**CG**	**EG1**	**EG2**	**EG3**
**Ingredients (%)**				
corn flour	52.0	52.0	52.0	50.0
soy flour	32.0	32.0	32.0	32.0
wheat flour	8.50	6.00	3.50	3.50
vegetable oil	2.50	2.50	2.50	2.50
gluten corn	2.00	2.00	2.00	2.00
GP	-	2.50	5.00	7.00
Minerals	3.00	3.00	3.00	3.00
**Chemical Composition (%)**[1]				
Dry matter	85.7	85.5	83.5	81.9
crude protein	24.3	24.1	24.5	25.1
lipid	6.53	6.52	6.53	6.55
crude fiber	5.25	5.22	6.04	6.76
starch	45.2	45.1	44.6	43.8
ash	6.81	6.77	7.20	7.60

[1] Data are reported on a dry matter (DM) basis. GP = grape pomace
