# Supplementary material for: Influence of Grape Pomace Intake on Nutritional Value, Lipid Oxidation and Volatile Profile of Poultry Meat
Source: Foods. 2020 Apr 17;9(4):508. doi: 10.3390/foods9040508 (PMC7230919; doi:10.3390/foods9040508)
Supplement: Supplementary file 1 [file foods-09-00508-s001.zip › Table S2.pdf]

Supplementary Table 2. Body weight (BW) and feed conversion ratio (FCR) evaluated after 1, 7, 14, 21, 28, 35, 42 and 49 days of age for chicken belonging to the control group (CG) and chicken fed the dietary grape pomace supplementation of 2.5 % (EG1), 5 % (EG2) and 7 % (EG3).

age (days)	Parameter	CG	EG1	EG2	EG3
1	BW[1]	53 ± 0.86	53 ± 1.13	54 ± 1.35	55 ± 2.53
	FCR	-	-	-	-
7	BW[1]	181.8 ± 10.2	183.5 ± 11.2	182.5 ± 12.2	183.9 ± 12.6
	FCR	0.887	0.869	0.881	0.885
14	BW[1]	472.7 ± 10.7	470.6 ± 17.1	471.1 ± 15.2	472.3 ± 13.8
	FCR	1.152	1.159	1.148	1.151
21	BW[1]	923.5 ± 16.9	921.7 ± 14.7	917.9 ± 16.4	919.6 ± 16.3
	FCR	1.306	1.311	1.308	1.307
28	BW[1]	1509 ± 76	1499 ± 81	1503 ± 73	1501 ± 79
	FCR	1.440	1.441	1.446	1.445
35	BW[1]	2177 ± 101	2172 ± 98	2167 ± 104	2171 ± 91
	FCR	1.568	1.576	1.569	1.572
42	BW[1]	2871 ± 112	2880 ± 145	2869 ± 102	2866 ± 110
	FCR	1.709	1.668	1.701	1.703
49	BW[1]	3581 ± 141	3529 ± 137	3601 ± 108	3578 ± 145
	FCR	1.807	1.832	1.814	1.797

[1] Data are reported as mean values (g) ± standard deviation (SD). BW = body weight; FCR = feed conversion ratio (cumulative feed intake / body weight).
